# The Relationship between Cognitive Functions and Psychopathological Symptoms in First Episode Psychosis and Chronic Schizophrenia

**DOI:** 10.3390/jcm11092619

**Published:** 2022-05-06

**Authors:** Katarzyna Rek-Owodziń, Ernest Tyburski, Piotr Plichta, Katarzyna Waszczuk, Maksymilian Bielecki, Krzysztof Wietrzyński, Piotr Podwalski, Krzysztof Rudkowski, Anna Michalczyk, Tomasz Grąźlewski, Leszek Sagan, Jolanta Kucharska-Mazur, Jerzy Samochowiec, Monika Mak

**Affiliations:** 1 Department of Health Psychology, Pomeranian Medical University in Szczecin, 71-457 Szczecin, Poland; ernest.tyburski@pum.edu.pl (E.T.); piotr.plichta@pum.edu.pl (P.P.); makrymilian.bielecki@pum.edu.pl (M.B.); krzysztofwietrzynski@gmail.com (K.W.); monika.mak@pum.edu.pl (M.M.); 2 Department of Psychiatry, Pomeranian Medical University in Szczecin, 71-457 Szczecin, Poland; zurawska1989@gmail.com (K.W.); piotr.podwalski@gmail.com (P.P.); krudkowski@gmail.com (K.R.); annakarolina6@wp.pl (A.M.); tgrazlewski@gmail.com (T.G.); jolanta.kucharska.mazur@pum.edu.pl (J.K.-M.); jerzy.samochowiec@pum.edu.pl (J.S.); 3 Department of Neurosurgery, Pomeranian Medical University in Szczecin, 71-252 Szczecin, Poland; leszekm.sagan@gmail.com

**Keywords:** first-episode psychosis, chronic schizophrenia, cognitive functions, psychopathology, MATRICS Consensus Cognitive Battery, MCCB, PANSS

## Abstract

Impairments in cognitive functions are one of the main features of schizophrenia. A variety of factors can influence the extent of cognitive deficits. In our study, we examined the severity of cognitive deficits at different stages of the disease and the relationship between psychopathological symptoms and cognitive functions. We recruited 32 patients with first-episode psychosis (FEP), 70 with chronic schizophrenia (CS), and 39 healthy controls (HC). Psychopathological symptoms were evaluated with the Positive and Negative Syndrome Scale (PANSS) and cognitive functions were measured with the MATRICS Cognitive Consensus Battery (MCCB). Cognitive deficits were present in both FEP and CS participants. CS individuals had lower overall scores and poorer working memory; however, clinical variables appeared to play a significant role in these scores. In FEP, disorganization correlated negatively with verbal and visual learning and memory, social cognition, and overall score; negative symptoms negatively correlated with social cognition. In CS participants, disorganization correlated negatively with speed of processing, reasoning, problem solving, and overall score; negative symptoms were negatively correlated with speed of processing, visual learning, memory, and overall score; positive symptoms were negatively correlated with reasoning and problem solving. Our findings indicate that psychopathological symptoms have a significant impact on cognitive functions in FEP and CS patients.

## 1. Introduction

Cognitive impairments are a core feature of schizophrenia that affect the overall functioning of patients. Various cognitive functions, including memory, verbal fluency, speed of processing, attention and executive function, are disrupted in patients with psychosis [1,2,3]. It is known that cognitive functioning may be impaired at different stages of psychosis [4,5]. Deficits of varying severity are present in patients with first-episode psychosis (FEP), chronic schizophrenia (CS) and even before the full manifestation of the illness in the ultra-high risk for psychosis state (UHR) [1,2]. The extent of cognitive impairment in schizophrenia ranges from 1 to 2.5 standard deviations below healthy control groups [6,7]. Intergroup studies have tried to determine if the severity of these disturbances changes with the duration of psychotic symptoms. Data regarding the progression of deficits in cognitive functioning at different stages of the psychosis spectrum are inconsistent. While some studies have found that deficits in different cognitive domains—such as global intelligence, attention, verbal learning, speed of processing, problem solving, and verbal and visual memory—are deeper in CS than in FEP patients [4,5], other studies indicate that cognitive functions are impaired at the same level in both groups [1,3,8]. Despite the disagreements on the dynamics of these changes, cognitive impairments remain more stable during a patient’s life than do positive and negative symptoms of schizophrenia [9].

Much research has been conducted to establish the direction of the relationship between the symptoms of schizophrenia and cognitive deficits. The debate over whether the neurodevelopmental or neurodegenerative model better explains the course of cognitive disorders is still relevant. Patients diagnosed with schizophrenia often present some cognitive impairments in late childhood or adolescence [1,7]. Neuroimaging studies reveal specific structural and/or functional brain changes related to cognitive deficits in patients with schizophrenia, such as gray matter abnormalities, cingulum bundle integrity disruptions, and decreased activity in the left thalamus and inferior/posterior cortical areas [10,11]. It is still unclear whether these changes are already present before the development of the illness or if they appear as a result of the neurodegenerative processes of psychosis. 

Knowledge about the relationship between cognitive impairments and the psychopathology symptoms of psychoses is very important, as it can suggest directions for diagnosis and treatment. Studies indicate that disturbances in cognitive functions are associated with clinical outcomes in schizophrenia [5,12]. Bozikas et al. [13] have demonstrated the relationship between severity of negative symptoms and impairments in cognitive functions such as semantic memory, verbal memory and auditory attention. A systematic review of the literature indicates that there is an association between the negative and disorganized symptoms and cognitive deficits [14]. This association has not been found regarding positive symptoms [14].

A variety of batteries are used in studies investigating cognitive functioning in FEP and CS patients but recently, the Measurement and Treatment Research to Improve Cognition in Schizophrenia (MATRICS) has become popular. This neuropsychological battery is used internationally, as it is a well standardized method of measuring cognitive impairments in schizophrenia with a high test–retest reliability and relationship to functional outcomes [15]. The MATRICS Consensus Cognitive Battery (MCCB) uses seven domains to measure cognitive functioning: working memory, attention/vigilance, verbal learning and memory, visual learning and memory, reasoning and problem solving, speed of processing, and social cognition [15].

To our knowledge, this is the first study examining the relationship between psychopathological symptoms evaluated with the Positive and Negative Syndrome Scale (PANSS) and cognitive functions measured with the MCCB in FEP and CS groups in the European population [16,17]. A review of research on cross-cultural differences in schizophrenia shows that there are differences both in the course of the symptoms and in the performance of individuals based on cultural context. These differences were noticeable in terms of both positive and negative symptoms. For this reason, it seems particularly useful to conduct a study on a European population [18]. Three earlier studies comparing cognitive impairments measured with the MCCB and psychopathological symptoms measured by PANSS have been conducted in China [3,5,19]. All these studies showed a deterioration in cognitive performance in FEP and CS compared to HC and found different correlations between cognitive performance and psychopathology symptoms [3,5,19]. Liu et al. [3] found a negative correlation between negative symptoms and attention, visual learning and reasoning, as well as between PANSS total score and visual learning in schizophrenia patients. Negative correlations of MCCB total score with PANSS total score, negative symptoms and general psychopathology symptoms have been found by Wu et al. [5]. Yang et al. [19] indicated that there are negative correlations of positive symptoms with verbal learning, of negative symptoms with speed of processing, verbal learning and reasoning, and of PANSS total score with verbal learning and visual learning. Previous research focused on three or four factors measured by the PANSS (total score, positive symptoms, negative symptoms, and general psychopathology). Meta-analysis of the structure of the PANSS indicates that there are five factors (positive symptoms, negative symptoms, disorganization, affect, and resistance) that are extremely useful in the assessment of the severity of symptoms [20]. In our study, we compared MCCB scores with five psychopathological factors measured by PANSS in FEP and CS patients to assess whether there is a relationship between impairments of cognitive functions and severity and duration of psychopathological symptoms.

## 2. Materials and Methods

### 2.1. Participants

A total of 102 patients, including 32 with first-episode psychosis (FEP) and 70 chronic schizophrenia (CS) patients, were recruited from the inpatients, day treatment patients, and outpatients at the Clinic of Psychiatry at the Pomeranian Medical University in Szczecin, Poland. All participants gave their written consent before participating in our study.

First-episode psychosis patients had to meet the following criteria: (1) experiencing a current acute psychotic episode diagnosed by a psychiatrist with the International Statistical Classification of Diseases and Related Health Problems (ICD-10) [21] and the Mini-International Neuropsychiatric Interview (MINI) [22]; (2) no prior psychotic episode; (3) no prior antipsychotic treatment for longer than 4 weeks; (4) being aged 18–40; and (5) being of European ethnicity. The exclusion criteria were the presence of: (1) any relationship between current symptoms and substance use; (2) substance use disorder; (3) other mental or neurological diseases; or (4) severe somatic diseases.

Chronic schizophrenia patients had to meet the following criteria: (1) a diagnosis of schizophrenia made by a psychiatrist based on the International Statistical Classification of Diseases and Related Health Problems (ICD-10) and the Mini-International Neuropsychiatric Interview (MINI); (2) at least 10 years duration of symptoms; (3) being aged 18–60; and (4) being of European ethnicity. The exclusion criteria were the presence of: (1) a substance use disorder; (2) any other mental or neurological disease; (3) severe somatic diseases; (4) any other mental or neurological disease; and (5) severe somatic diseases.

The healthy control group consisted of 39 persons without mental or neurological disorders. They were matched with the clinical groups for age, sex, and number of years of education. They were recruited from the local community. They were assessed by a psychiatrist with a structured interview. The inclusion criteria were: (1) being aged 18–60; (2) being of European ethnicity; and (3) having no prior mental disorder. Exclusion criteria for healthy controls were the same as those for patients.

### 2.2. Clinical Measures

Structured diagnostic interviews were conducted by licensed psychiatrists. We gathered data on general and sociodemographic characteristics, medical status, basic family history, and current complaints. Mental status was evaluated with the Mini-International Neuropsychiatric Interview (MINI). To measure the severity of psychopathological symptoms in FEP and CS patients, we used the Positive and Negative Syndrome Scale (PANSS). In analysis, we distinguished five psychopathological dimensions: negative, positive, disorganized, excited, and anxiety and depression, as recommended by Shafer et al. [20] based on their meta-analysis of 45 factor analyses of PANSS. We assessed severity of schizophrenia and its impact on functioning using the Global Assessment of Functioning (GAF) [23].

### 2.3. Neurocognitive Assessment

Neurocognitive assessments were conducted by well-qualified psychologists. Cognitive functioning was evaluated with the Polish version of the MATRICS Consensus Cognitive Battery (MCCB) [17,24]. Table 1 shows the seven domains and their respective tests on the MCCB. We also calculated a global composite score for each patient.

### 2.4. Statistical Analysis

Statistical analysis of the results was performed using IBM SPSS 27 (IBM Corp., Redmont, VA, USA). Continuous variables were presented as means (*M*) and standard deviations (*SD*). The normality of the distributions were examined with the Shapiro–Wilk test as well as skewness and kurtosis values. We assumed that skewness values from −2 to +2 and kurtosis values from −7 to +7 indicated normal distributions of variables [25]. Age, years of education, and scores from the MCCB battery for all cognitive domains and overall score were normally distributed in all three groups. Chlorpromazine equivalent and global functioning on the GAF were normally distributed in both clinical groups, but illness duration and exacerbation were not normally distributed. Moreover, psychopathological dimensions assessed by PANSS were normally distributed only in the FEP group. Differences between the two groups were examined with the Student’s *t* test (if the relevant assumptions were met) and the Mann–Whitney *U* test (if the relevant assumptions were not met). Differences between three groups in cognitive domains and overall score were examined with analysis of covariance (ANCOVA), controlling for the effect of age and years of education. Moreover, in the case of significant differences in cognitive functions between the two clinical groups, we conducted an ANCOVA to control for the effects of duration of illness, exacerbations (defined as the number of acute exacerbations of psychotic symptoms in the course of the illness), chlorpromazine equivalent, and psychopathological dimensions (positive symptoms, disorganization, and resistance). Comparisons between groups were performed using the Games–Howell or Bonferroni post hoc test (for parametric tests). Cohen’s *d* and η^2^ (parametric tests) [26] or Wendt’s *r_U_* (non-parametric tests) [27] were used to determine the magnitudes of effect sizes for differences between groups. Finally, in order to assess the relationships between psychopathological symptoms and cognitive domains in both clinical groups, Spearman’s *r* or Spearman’s *rho* correlation coefficients were estimated. Single stepwise regression (for one psychopathological symptom as predictor and cognitive ability as a single dependent variable) or multivariate stepwise regression (for two psychopathological symptoms as predictors and cognitive ability as a single dependent variable) was performed for bivariate correlations in clinical groups that appeared to be significant. We used a stepwise regression model to find the strongest predictor of cognitive abilities in study groups [28]. The variance inflation factor (VIF: >10) was calculated to assess multicollinearity. Holm–Bonferroni *p*-value correction was used for all statistical analyses (multiple comparisons and correlations). The alpha criterion level was set at 0.05 and all statistical analyses had a statistical power greater than 0.80 [26].

## 3. Results

### 3.1. Participant Characteristics

Demographic and clinical characteristics are presented in Table 2. There were no significant differences in sex; however, the groups differed significantly in age (*p* < 0.001) and in years of education (*p* = 0.025). Post hoc analyses showed that patients with FES were younger (*p* < 0.001 and *p* = 0.001) than patients with CS and HC, and patients with CS had fewer years of education (*p* = 0.034) than HC. After Holm–Bonferroni *p*-value correction, patients with FEP had shorter duration of illness (*p* < 0.001), fewer exacerbations (*p* < 0.001) and less chlorpromazine equivalent (*p* = 0.006) than CS patients, and greater severity of positive symptoms (*p* < 0.001), disorganization (*p* = 0.012) and resistance (*p* = 0.045), as measured by PANSS. The clinical groups did not significantly differ in antipsychotic medications, global functioning measured by GAF, negative symptoms, or affect as assessed by PANSS.

### 3.2. Differences in Cognitive Functions

**Figure 1:**
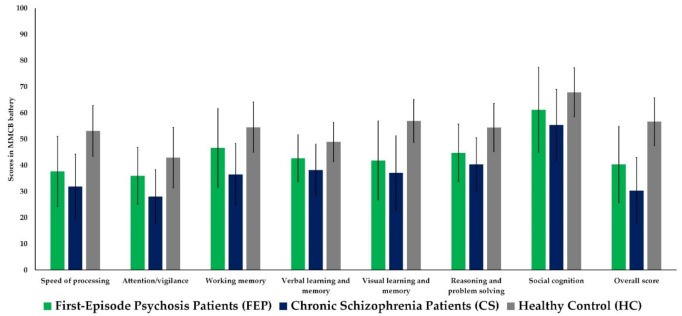
Profile of comparison of cognitive domains measured by MMCB battery between participants from three groups. MMCB = MATRICS Consensus Cognitive Battery.

As can be seen in Table 3 and Figure 1, there were significant differences in all cognitive domains (*p* < 0.001) and overall score (*p* < 0.001) between all groups after adjusting for age and years of education. Post hoc analysis showed that FEP patients had lower scores than HC in all cognitive domains (0.05 > *p* < 0.001) and lower overall score (*p* < 0.001). Moreover, FEP patients had higher results for working memory (*p* < 0.01) and overall score (*p* < 0.05) than CS patients. CS patients had lower results in all cognitive domains (*p* < 0.001) and in overall score (*p* < 0.001) than HC.

Further analysis showed that there were no significant differences in working memory and overall score between the two clinical groups after adjusting for clinical variables (duration of illness, exacerbations, chlorpromazine equivalent and psychopathological dimensions).

### 3.3. Relationship between Psychopathological Dimensions and Cognitive Functions

**Figure 2:**
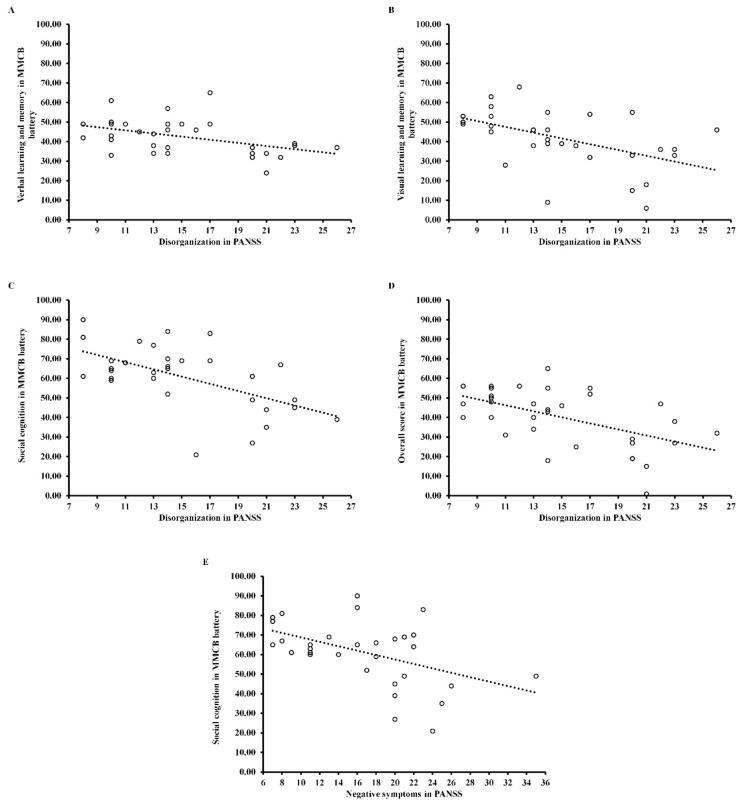
Scattergram for the relationship between psychopathological dimensions assessed by PANSS ((**A**–**D**) for disorganization and (**E**) for negative symptoms) and cognitive domains measured by MMCB in First-Episode Psychosis Patients (FEP). MMCB = MATRICS Consensus Cognitive Battery.

**Figure 3:**
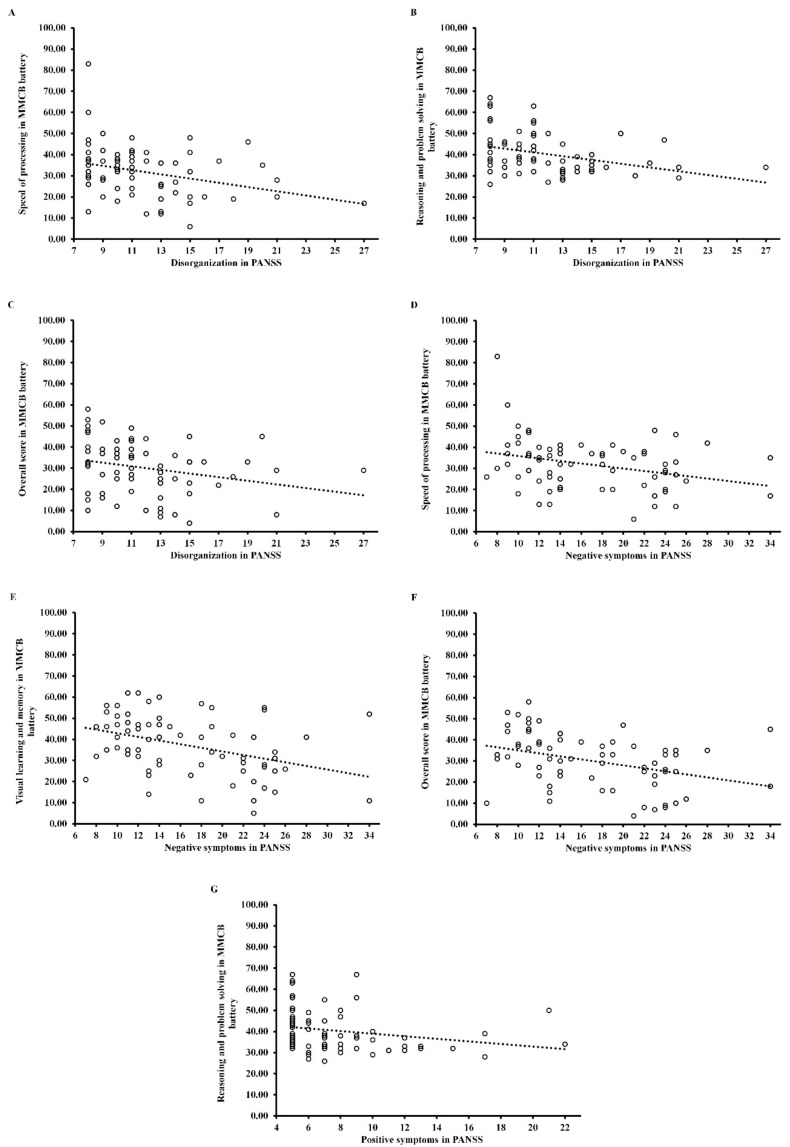
Scattergram for the relationship between psychopathological dimensions assessed by PANSS ((**A**–**C**) for disorganization, (**D**–**F**) for negative symptoms, and (**G**) for positive symptoms) and cognitive domains measured by MMCB in Chronic Schizophrenia Patients (CS). MMCB = MATRICS Consensus Cognitive Battery.

As can be seen in Table 4 and Figure 2, disorganization correlated negatively with verbal learning and memory (*r* = −0.45; *p* < 0.05), visual learning and memory (*r* = −0.50; *p* < 0.05), social cognition (*r* = −0.58; *p* < 0.001) and overall score (*r* = −0.54; *p* < 0.05) in FEP patients. Moreover, in this group negative symptoms correlated negatively with social cognition (*r* = −0.47; *p* < 0.05). In CS patients (Table 4 and Figure 3), disorganization negatively correlated with speed of processing (*rho* = −0.32; *p* < 0.05), reasoning and problem solving (*rho* = −0.36; *p* < 0.05) and overall score (*rho* = −0.34; *p* < 0.05). Moreover, negative symptoms negatively correlated with speed of processing (*rho* = −0.30; *p* < 0.05), visual learning and memory (*rho* = −0.39; *p* < 0.05), and overall score (*rho* = −0.39; *p* < 0.001), while positive symptoms negatively correlated with reasoning and problem solving (*rho* = −0.34; *p* < 0.05). All correlation coefficients have been corrected (Holm–Bonferroni *p*-value correction).

In patients with FEP, disorganization was significant predictor of speed of processing (β = −0.31; *t* = −2.70; *p* = 0.009), predicting about 8% of the variance (the model was well suited to the analyzed data, *F* = 7.31; *p* = 0.009). Negative symptoms introduced into the model were statistically excluded. In this group, disorganization was also a significant predictor of verbal learning and memory (β = −0.45; *t* = −2.77; *p* = 0.010), visual learning and memory (β = −0.50; *t* = −3.15; *p* = 0.040), and overall score (β = −0.54; *t* = −3.45; *p* = 0.002), predicting about 18% of the variance (the model was well suited to the analyzed data, *F* = 7.66; *p* = 0.010), 22% of the variance (the model was well suited to the analyzed data, *F* = 9.89; *p* = 0.004) and 26% of the variance (the model was well suited to the analyzed data, *F* = 12.04; *p* = 0.002), respectively.

In patients with CS, disorganization was a significant predictor of social cognition (β = −0.58; *t* = −3.87; *p* < 0.001), predicting about 31% of the variance (the model was well suited to the analyzed data, *F* = 14.95; *p* < 0.001). The negative symptoms variable introduced into the model was statistically excluded. Moreover, disorganization was a significant predictor of reasoning and problem solving (β = −0.34; *t* = −2.95; *p* = 0.004), predicting about 10% of the variance (the model was well suited to the analyzed data, *F* = 8.71; *p* = 0.004). The positive symptoms variable introduced into the model was statistically excluded. In this group, the negative symptoms variable was also a significant predictor of visual learning and memory (β = −0.39; *t* = −3.49; *p* < 0.001) predicting about 14% of the variance (the model was well suited to the analyzed data, *F* = 12.19; *p* < 0.001). Moreover, the negative symptoms variable was a significant predictor of overall score (β = −0.39; *t* = −3.49; *p* < 0.001), predicting about 12% of the variance (the model was well suited to the analyzed data, *F* = 12.19; *p* < 0.001). The disorganization symptoms variable introduced into the model was statistically excluded.

## 4. Discussion

In this study we investigated differences in cognitive functions between psychotic patients at different stages of the illness compared to healthy controls and the relationship between psychopathology and cognitive impairments in the clinical group. The results of our study showed that, compared to healthy controls (HC) and controlling for covariates, first-episode psychosis (FEP) and chronic schizophrenia (CS) patients presented impairments in cognitive functions and scored significantly lower in all cognitive domains as measured by the MATRICS Consensus Cognitive Battery (MCCB; Table 3). There were no significant differences between the studied groups in terms of sex. Despite there being some differences in terms of age and years of education (FEP patients were younger and CS patients had fewer years of education) cognitive deficits were closely related to the psychotic process. Then, when we compared FEP and CS patients’ scores, the results showed that CS patients performed worse on tests measuring working memory and got overall lower scores than FEP patients. Intergroup differences in terms of age resulted from the inclusion criteria for the study group. These criteria were defined in this way due to the nature of the studied groups. The first psychotic episode most often occurs in early adulthood, hence the age restriction in this group. In the case of CS, the inclusion criterion was a minimum 10-year duration of the disease, therefore the people recruited to this group could be older than in the FEP group.

Our results regarding cognitive impairments in FEP and CS groups are in line with previous studies [5,8,29,30,31], which found that both FEP and CS patients performed worse than HC in all cognitive domains measured by the MCCB (attention/vigilance, reasoning and problem solving, social cognition, speed of processing, working memory, and verbal and visual learning) and in overall score. This confirms that cognitive dysfunction is one of the central symptoms of psychotic disorders. These results can be considered consistent with the neurodevelopmental model of schizophrenia, since the presence of cognitive disorders at various stages of psychosis suggests the initial presence of these deficits. Additionally, these assumptions are supported by reports on the stability of cognitive deficits in schizophrenia [4]. Moreover, some studies have found that UHR patients also get significantly lower cognitive scores in MCCB than HC, which also supports the validity of the neurodevelopmental model of psychosis [3,29]. Some of the previous studies did not find that significant differences apply to all cognitive domains measured with the MCCB. Results published by Liu et al. [3] show that there are no differences in spatial working memory between FEP and HC. Yang et al. [19] did not confirm the presence of differences in attention/vigilance, spatial working memory, verbal learning, and social cognition between FEP, CS and HC. In both of these studies, differences were present in the remaining domains. The analysis of the results of our study shows significant differences in cognitive functioning in FEP and CS patients compared to HC, with the normal distribution of results in the studied groups additionally implying that the MCCB is a reliable tool for the assessment of cognitive functions in the European population.

Deficits in working memory are considered to be a core cognitive impairment in psychotic patients. The first study using MCCB in which FEP patients were recruited is in line with our results, suggesting that deficits in working memory progress over the course of the illness and are deeper in CS than in FEP patients [30]. That study found a similar relationship in social cognition, which our study did not confirm [30]. Wu et al. [5] found that CS patients performed worse on a digital sequence task involving working memory. Other studies that have used MCCB in FP and CS patients found no significant differences in working memory [3,8,19,29,31]. A study conducted on the Spanish population indicates that FEP patients perform better on verbal and visual learning and worse on attention than CS patients [31]. Corigliano et al. [29] on the other hand reports that FEP patients perform better on tasks involving attention than CS. To our knowledge, only the results of one study in which FEP were compared to CS on MCCB scores were in line with ours, with CS patients having worse total scores than FP. These reports prompt reflection on the validity of the neurodegenerative theory of schizophrenia. While some studies show a progression of cognitive deficits in CS vs. FEP, others deny it [3,4,8,19]. Therefore, we should consider whether some other factors influence the severity of cognitive deficits in these groups. In our further statistical analysis, we added clinical variables (duration of illness, exacerbations, chlorpromazine equivalent, and psychopathological dimensions) as covariates and this substantially changed our results, showing that the differences between cognitive performance in FEP and CS were not significant. These results indicate the stability of cognitive deficits in patients with FEP and CS and emphasize the importance of controlling variables such as clinical symptoms or disease duration in statistical analyses. 

Both correlation and regression analysis of our results revealed a relationship between some psychopathological symptoms measured with PANSS and cognitive domains in the FEP and CS groups (*p* < 0.05 or *p* < 0.001; Table 4). In the FEP group, disorganization negatively correlated with both verbal and visual learning and memory, social cognition and overall score. In that group, negative symptoms negatively correlated with social cognition. In CS patients, disorganization negatively correlated with speed of processing, reasoning and problem solving, and overall score; negative symptoms negatively correlated with speed of processing, visual learning, memory, and overall score; and positive symptoms negatively correlated with reasoning and problem solving. These findings imply that psychopathology symptoms are closely related to cognitive impairments at different stages of psychosis. In both FP and CS groups, disorganization and negative symptoms were correlated with some cognitive functions. We are aware of three studies demonstrating that negative symptoms measured with PANSS are associated with deterioration in cognitive function assessed with MCCB [3,5,19]. The study conducted by Bagney et al. [32] did not find correlations between negative symptoms and cognitive functioning. It is worth noting that the aforementioned study is the only one in which the five factor model of PANSS was used. In it, the relationship between MCCB performance was associated only with disorganization [32]. This is a response to doubts about the significant impact of negative symptoms on cognition. The previous classification of symptoms in the PANSS scale added issues related to cognitive functions (disorganization) to the group of negative symptoms. The disorganization factor is generally identified with disturbances of the form of thought, poverty of content of speech, and disturbance in affective reactions (inappropriate affect) [33]. Disturbances in the form of thoughts are manifested as difficulties with abstracting, using metaphors, generalizing, searching for antonyms, a tendency towards incoherence, tangentiality, loosening of associations, derailment, etc. Poverty of content of speech is associated with limited expression and difficulty formulating statements due to difficulties in thinking. Inappropriate affect is associated with expression with the use of facial expressions, gestures and body language incongruent with what is being said. In the Positive and Negative Syndrome Scale, “formal thought disorders”, “difficulties in abstract thinking”, and “lack of attention” are the core items of a disorganization factor [20]. The disorganization factor has some conceptual overlap with cognitive performance, such as attention, perception, or abstraction [33]. Some earlier studies on the relationship between disorganization symptoms and cognitive performance, using methods different from those in our study, indicated a significant relationship between the severity of symptoms of disorganization and the deterioration of cognitive performance [34,35]. One of the studies showed that the effect size of the relationship between neurocognition and disorganization was significantly larger than between neurocognition and positive symptoms (reality distortion) [34]. Another study found that inverse associations with neurocognition and social cognition were significantly greater for disorganized than negative symptoms [35]. As some reports did not show a significant relationship between the symptoms of dysregulation and the efficiency of cognitive processes, the continuation of research in this area, including the results of our study, is particularly important from the perspective of understanding the relationship between psychopathological symptoms and cognitive processes in patients with a psychosis spectrum [33]. Despite the fact that in our study we observed a relationship between negative symptoms and cognitive deficits in the FP and CS groups, this relationship was more frequent in relation to disorganization symptoms. Regarding the MCCB overall score, disorganization symptoms in FEP patients and negative symptoms in CS patients predicted outcomes. This may suggest that negative symptoms affect cognition more in the later stages of the disease, and disorganization throughout the course of the disease.

The strength of our study is that we compared two clinical groups from different points of the schizophrenia spectrum using the same neurocognitive battery. We also consider the use of MCCB as a strong point of this study, because it is a consistent battery of tests that reliably measures the tested functions. Moreover, applying the five-factor PANSS model in accordance with the latest research reports is the third strength of the study.

This work has several limitations. First of all, our study has a cross-sectional design. This kind of study cannot control for potential cohort effects. Longitudinal studies have many benefits in describing differences between clinical samples, as in this kind of study design we can observe changes over time in the same participants. A second limitation is that the chronic patients had a longer duration of illness, significantly more exacerbations, and higher chlorpromazine equivalent. However, this situation is very hard to avoid since duration of illness is the factor that differentiates first episode psychosis from chronic schizophrenia, and it affects the number of exacerbations and the amount of medications used in treatment. Third, FEP patients had higher scores on three PANSS dimensions: positive symptoms, disorganization, and resistance. In future studies it would be useful to select clinical groups based on the severity of psychopathological symptoms. Even though we included clinical symptoms as covariates in our analysis, we advise caution when interpreting our data. Fourth, the study groups were unequal: the group of CS patients was larger than the FEP and HC groups. Other, similar studies in patients with FEP and CS were either conducted with fewer subjects, or also included a larger sample of patients with CS than with FEP [3,4,5,19]. It can be assumed that the greater number of subjects in the FEP and HC groups could contribute to greater statistical power of the results. In the future, we plan to extend this study to a larger study group.

## 5. Conclusions

To conclude, our study showed that cognitive deficits are present in both FEP and CS patients. The general severity of these deficits, with particular emphasis on working memory, turned out to be greater in the CS group; however, a significant role in their occurrence is attributed to clinical variables. Psychopathological symptoms are a significant factor influencing the presence of cognitive impairments. We observed that negative and dysregulation symptoms had the greatest effect on cognitive performance. Dysregulation symptoms in FEP patients and negative symptoms in CS patients were predictors of overall MCCB score.

## Figures and Tables

**Table 1:** Cognitive domains and cognitive tests from the MCCB [17,24].

Cognitive Domain	Cognitive Tests
Attention/vigilance	Continuous Performance Test—Identical Pairs (CPT-IP)
Reasoning and problem solving	Neuropsychological Assessment Battery (NAB): Mazes
Social cognition	Mayer–Salovey–Caruso Emotional Intelligence Test (MSCEIT): Managing Emotions
Speed of processing	Trail Making Test (TMT): Part A
	Brief assessment of Cognition in Schizophrenia (BACS)
	Category Fluency: Animal Fluency
Working memory	Wechsler Memory Scale (WMS^®^-III): Spatial Span forward and backward (WMS-SS)
	Letter number span (LNS)
Verbal learning and memory	Hopkins Verbal Learning Test—Revised (HVLTR)
Visual learning and memory	Brief Visuospatial Memory Test—Revised (BVMT-R)

MMCB = MATRICS Consensus Cognitive Battery.

**Table 2:** Demographic and clinical characteristics of participants from three groups.

	First-Episode Psychosis Patients (FEP) (*n* = 32)	Chronic Schizophrenia Patients (CS) (*n* = 70)	Healthy Control (HC) (*n* = 39)	*F*/*χ*^2^/*t*/*Z*	η^2^/*d*/*r_U_*
Age: *M* (*SD*)	28.09 (6.32) ^e^***^, g^***	39.10 (6.85)	37.08 (7.94)	27.29 ^a^***	0.28 ^h^
Years of education: *M* (*SD*)	12.81 (3.52)	13.20 (2.93) ^f^*	14.59 (2.62)	3.77 ^a^*	0.05 ^h^
Sex: female/male	18/14	30/40	23/16	3.18 ^b^	-
Antipsychotic medications:					
Atypical: *n* (%)	28 (87.50)	46 (65.71)	-	6.91 ^b^	-
Atypical and typical: *n* (%)	2 (6.25)	20 (28.57)	-		
Typical: *n* (%)	1 (3.125)	3 (4.29)	-		
No medications: *n* (%)	1 (3.125)	1 (1.43)	-		
Chlorpromazine equivalent (mg): *M* (*SD*)	478.69 (299.48)	671.69 (290.30)	-	−3.08 ^c^**	0.62 ^i^
Duration of illness in years: *M* (*SD*)	0.71 (1.27)	14.97 (5.49)	-	−8.13 ^d^***	1.00 ^j^
Exacerbation: *M* (*SD*)	1.06 (0.25)	6.19 (4.30)	-	−8.06 ^d^***	0.98 ^j^
Global functioning in GAF: *M* (*SD*)	57.61 (17.04)	55.51 (14.91)	-	0.62 ^c^	-
Psychopathological dimensions in PANSS:					
Positive symptoms: *M* (*SD*)	12.03 (4.80)	7.71 (3.77)	-	−4.48 ^d^***	0.55 ^j^
Negative symptoms: *M* (*SD*)	16.81 (6.69)	16.77 (6.45)	-	−0.10 ^d^	-
Disorganization: *M* (*SD*)	14.91 (5.06)	11.81 (3.81)	-	−3.02 ^d^*	0.37 ^j^
Affect: *M* (*SD*)	10.19 (3.88)	8.79 (3.38)	-	−1.78 ^d^	-
Resistance: *M* (*SD*)	5.38 (1.91)	4.66 (1.99)	-	−2.43 ^d^*	0.25 ^j^

GAF = Global Assessment of Functioning. PANSS = Positive and Negative Syndrome Scale. ^a^ One-way analysis of variance *F* test. ^b^ Chi-squared test. ^c^ Student’s *t* test. ^d^ Mann-Whitney *U* test. All *p*-values for post hoc of ANOVA. ^e^ FEP patients vs. HC participants. ^f^ CS patients vs. HC participants. ^g^ FEP patients vs. CS patients. ^h^ Eta squared effect size: small (0.01–0.059), medium (0.06–0.139), large (0.14–1.00). ^i^ Cohen’s *d* effect size: small (0.20–0.49), medium (0.50–0.79), large (> 0.80). ^j^ Wendt’s *r* rank-biserial correlation effect size: small (0.10–0.29), medium (0.30–0.49), large (> 0.50). * *p* < 0.05. ** *p* < 0.01. *** *p* < 0.001 (for ANOVA and after Holm-Bonferroni *p*-value correction for Student’s *t* test and Mann-Whitney *U* test).

**Table 3:** Comparison of cognitive domains measured by MMCB battery between participants from three groups.

	First-Episode Psychosis Patients (FEP) (*n* = 32)	Chronic Schizophrenia Patients (CS) (*n* = 70)	Healthy Control (HC) (*n* = 39)	*F*	η^2^
Speed of processing	37.66 (13.35) ^a^***	31.90 (12.27) ^b^***	53.15 (9.66)	39.86 ***	0.37
Attention/vigilance	35.97 (10.80) ^a^***	28.04 (10.24) ^b^***	42.90 (11.55)	23.53 ***	0.26
Working memory	46.59 (15.07) ^a^*^,c^**	36.54 (11.83) ^b^***	54.51 (9.57)	24.98 ***	0.27
Verbal learning and memory	42.69 (9.04) ^a^**	38.16 (9.92) ^b^***	48.95 (7.50)	15.31 ***	0.18
Visual learning and memory	41.84 (15.06) ^a^**	37.07 (14.20) ^b^***	56.90 (8.21)	26.38 ***	0.28
Reasoning and problem solving	44.75 (10.98) ^a^**	40.37 (10.08) ^b^***	54.44 (9.20)	21.28 ***	0.24
Social cognition	61.13 (16.18) ^a^*	55.40 (13.56) ^b^***	67.87 (9.33)	8.15 ***	0.11
Overall score	40.28 (14.64) ^a^***^, c^*	30.26 (12.68) ^b^***	56.69 (9.13)	50.57 ***	0.43

MMCB = MATRICS Consensus Cognitive Battery. *F* = Analysis of covariance *F* test. η^2^ = Eta squared effect size: small (0.01–0.059), medium (0.06–0.139), large (0.14–1.00). All *p*-values for ANCOVA were after co-variates controlling demographic variables (age and years of education). ^a^ FEP patients vs. HC participants. ^b^ CS patients vs. HC participants. ^c^ FEP patients vs. CS patients. * *p* < 0.05. ** *p* < 0.01. *** *p* < 0.001.

**Table 4:** Relationship between psychopathological dimensions assessed by PANSS and cognitive domains measured by MMCB battery in two clinical groups.

	Speed of Processing	Attention/Vigilance	Working Memory	Verbal Learning and Memory	Visual Learning and Memory	Reasoning and Problem Solving	Social Cognition	Overall Score
First-Episode Psychosis Patients (FEP)								
	*r*	*r*	*r*	*r*	*r*	*r*	*r*	*r*
Positive symptoms	−0.18	−0.05	−0.18	−0.27	−0.23	−0.16	−0.23	−0.28
Negative symptoms	−0.26	−0.05	−0.01	−0.05	−0.39	−0.26	−0.47 *	−0.35
Disorganization	−0.29	−0.31	−0.06	−0.45 *	−0.50 *	−0.32	−0.58 ***	−0.54 *
Affect	−0.10	0.40	0.02	0.20	0.07	0.15	−0.08	0.11
Resistance	−0.06	0.07	0.00	−0.22	−0.12	0.37	0.10	0.03
Chronic Schizophrenia Patients (CS)								
	*rho*	*rho*	*rho*	*rho*	*rho*	*rho*	*rho*	*rho*
Positive symptoms	−0.11	−0.07	−0.11	−0.01	−0.21	−0.34 *	−0.11	−0.22
Negative symptoms	−0.30 *	−0.20	−0.28	−0.14	−0.39 *	−0.16	−0.14	−0.39 ***
Disorganization	−0.32 *	−0.05	−0.20	0.01	−0.25	−0.36 *	−0.13	−0.34 *
Affect	0.02	−0.19	−0.01	0.12	−0.04	0.00	0.05	−0.01
Resistance	−0.25	0.10	0.06	0.03	−0.08	−0.26	0.00	−0.11

PANSS = Positive and Negative Syndrome Scale. MMCB = MATRICS Consensus Cognitive Battery. * *p* < 0.05. *** *p* < 0.001 (after Holm-Bonferroni *p*-value correction).

## Data Availability

Materials of the study reported here are available from the corresponding author on reasonable request.
